# MRI Radiomics Features From Infarction and Cerebrospinal Fluid for Prediction of Cerebral Edema After Acute Ischemic Stroke

**DOI:** 10.3389/fnagi.2022.782036

**Published:** 2022-03-03

**Authors:** Liang Jiang, Chuanyang Zhang, Siyu Wang, Zhongping Ai, Tingwen Shen, Hong Zhang, Shaofeng Duan, Xindao Yin, Yu-Chen Chen

**Affiliations:** ^1^Department of Radiology, Nanjing First Hospital, Nanjing Medical University, Nanjing, China; ^2^Department of Radiology, Nanjing Gaochun People’s Hospital, Nanjing, China; ^3^Department of Radiology, Affiliated Jiangning Hospital of Nanjing Medical University, Nanjing, China; ^4^GE Healthcare, Precision Health Institution, Shanghai, China

**Keywords:** stroke, biomarker, neuroimaging, machine learning, cerebrospinal fluid

## Abstract

Neuroimaging biomarkers that predict the edema after acute stroke may help clinicians provide targeted therapies and minimize the risk of secondary injury. In this study, we applied pretherapy MRI radiomics features from infarction and cerebrospinal fluid (CSF) to predict edema after acute ischemic stroke. MRI data were obtained from a prospective, endovascular thrombectomy (EVT) cohort that included 389 patients with acute stroke from two centers (dataset 1, *n* = 292; dataset 2, *n* = 97), respectively. Patients were divided into edema group (brain swelling and midline shift) and non-edema group according to CT within 36 h after therapy. We extracted the imaging features of infarct area on diffusion weighted imaging (DWI) (abbreviated as DWI), CSF on fluid-attenuated inversion recovery (FLAIR) (CSF_FLAIR_) and CSF on DWI (CSF_DWI_), and selected the optimum features associated with edema for developing models in two forms of feature sets (DWI + CSF_FLAIR_ and DWI + CSF_DWI_) respectively. We developed seven ML models based on dataset 1 and identified the most stable model. External validations (dataset 2) of the developed stable model were performed. Prediction model performance was assessed using the area under the receiver operating characteristic curve (AUC). The Bayes model based on DWI + CSF_FLAIR_ and the RF model based on DWI + CSF_DWI_ had the best performances (DWI + CSF_FLAIR_: AUC, 0.86; accuracy, 0.85; recall, 0.88; DWI + CSF_DWI_: AUC, 0.86; accuracy, 0.84; recall, 0.84) and the most stability (RSD% in DWI + CSF_FLAIR_ AUC: 0.07, RSD% in DWI + CSF_DWI_ AUC: 0.09), respectively. External validation showed that the AUC of the Bayes model based on DWI + CSF_FLAIR_ was 0.84 with accuracy of 0.77 and area under precision-recall curve (auPRC) of 0.75, and the AUC of the RF model based on DWI + CSF_DWI_ was 0.83 with accuracy of 0.81 and the auPRC of 0.76. The MRI radiomics features from infarction and CSF may offer an effective imaging biomarker for predicting edema.

## Introduction

Stroke is the second leading cause of death and third leading cause of disability in adults worldwide (Campbell and Khatri, 2020; Powers, 2020). Cerebral edema, a detrimental complication of stroke, is associated with death and neurologic deterioration after hemispheric infarction (Silver et al., 1984; Bar and Biller, 2018). Cerebral edema does not appear in the lacunar ischemic stroke subtype, even if they have multiple clinically associated silent lacunes, but, on the contrary, it tends to appear in acute non-lacunar cerebral infarcts (Blanco-Rojas et al., 2013). The development of edema usually presents as abrupt mental status worsening 2 days or more after admission. Earlier prediction of brain swelling and of those at risk of deterioration could guide the clinical selection of targeted therapies to minimize the risk of edema, cerebral herniation and secondary injury in stroke patients, and the selection of candidates for aggressive surgical procedures.

Currently, the radiographic assessment of edema is mostly based on standard CT findings (Fabritius et al., 2017; Muscari et al., 2019; Dhar et al., 2020; Foroushani et al., 2020; Lietke et al., 2020). This approach is insensitive to lesion or edema volume measurements, especially stroke within 12–24 h. In addition, edema was primarily seen in brain regions that flow pathways for cerebrospinal fluid (CSF)-interstitial fluid (ISF), and CSF is thought to be the primary source of early edema fluid after ischemic stroke (Lempriere, 2020; Mestre et al., 2020). Therefore, previous studies have shown that a reduction in CSF volume (ΔCSF) from baseline to 24 h CT is a promising early biomarker for worse neurologic outcome (Dhar, 2020; Dhar et al., 2020). Assessing edema utilizing only a reduction in CSF volume does not account for the influence of infarct volume on edema. Furthermore, lesion volume (even on MRI scans) only partially predicts the risk of herniation due to the edema that contributes to swelling and the risk of herniation (Strbian et al., 2013; Yoo et al., 2013). Machine learning (ML) algorithms are able to process high dimensional input data efficiently (Heo et al., 2020; Sirsat et al., 2020; Van Timmeren et al., 2020; Hamann et al., 2021). Many studies have shown that ML play an important role in stroke diagnosis, treatment and outcome (Heo et al., 2019; Fang et al., 2020; Kamel et al., 2020; Sirsat et al., 2020; Hamann et al., 2021). However, ML derived diffusion weighted imaging (DWI) imaging features for predicting edema has not been reported.

We aimed to develop and validate a stable ML model for predicting cerebral edema using a combination of imaging features of DWI infarct area and CSF on DWI or fluid-attenuated inversion recovery (FLAIR). Our hypothesis was that MRI radiomics features from infarction and CSF would improve the detection of patients developing edema.

## Materials and Methods

### Patients Selection and Clinical Data

**FIGURE 1:**
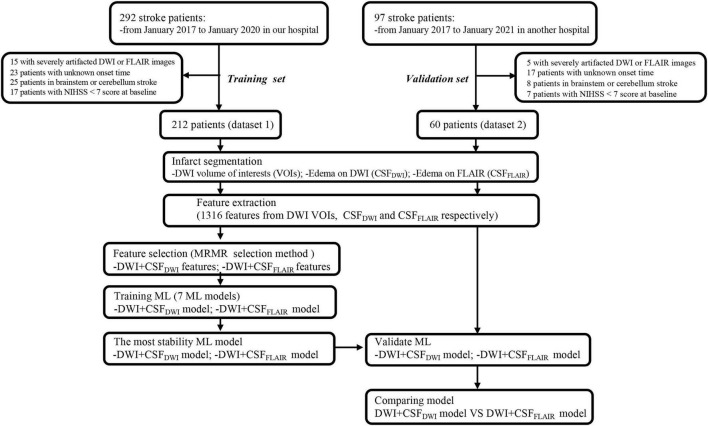
The flowchart of this study.

This was a retrospectively study using a prospective cohort that enrolled acute ischemic stroke (AIS) patients from Nanjing First Hospital (dataset 1) and Affiliated Jiangning Hospital of Nanjing Medical University (dataset 2) between January 2017 and January 2020. All patients provided informed consent for acute stroke imaging. Patients with AIS included in this study if (1) the onset of their first-ever stroke had occurred within the past 24 h, (2) MRI examinations were performed before endovascular thrombectomy (EVT) therapy, (3) patients underwent EVT therapy or bridging therapy (both intravenous thrombolysis (IVT) and EVT) according to the guidelines for managing AIS, and (4) patients underwent non-contrast CT within 36 h after EVT therapy. Patients for whom the stroke onset time was unknown, the final diagnosis was not stroke, or with stroke located in the brainstem or cerebellum were excluded. To analyze those at highest risk for edema, we excluded those with National Institutes of Health Stroke Scale (NIHSS) < 7 scores at baseline (Thoren et al., 2017). A total of 212 patients from dataset 1 and 60 patients from dataset 2 were included. Dataset 1 was used to train the models and to compare the performance of the different models so that the model which had the most stable and best performance could be selected. Dataset 2 was preserved as an independent external validation set. The flowchart of this study is shown in Figure 1.

Age, sex, NIHSS score on admission, time from symptom onset to MRI, time from symptom onset to intravenous thrombolysis (IVT), time from symptom onset to EVT, history of hypertension, diabetes mellitus, hyperlipidemia, and atrial fibrillation were recorded. Cerebral edema was defined as imaging evidence of brain swelling, and midline shift at the septum pellucidum or pineal gland on non-contrast CT within 36 h after EVT therapy (Wu et al., 2018). The study was approved by the local Ethics Committee of Nanjing Medical University.

### Volume of Interest Segmentation

**FIGURE 2:**
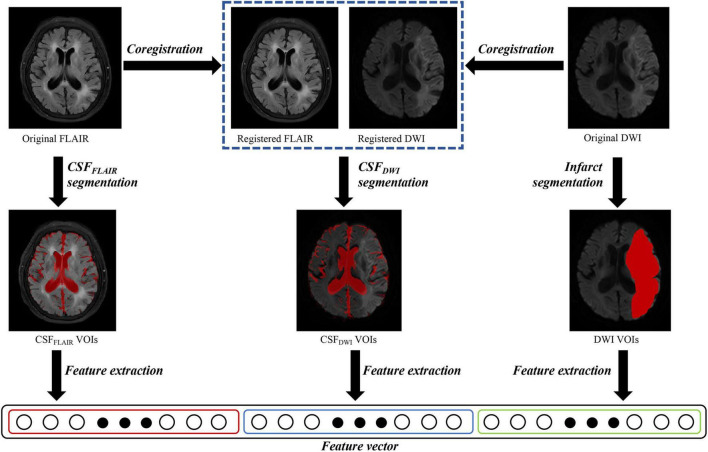
Overview of image processing with diffusion weighted imaging (DWI) and fluid attenuated inversion recovery (FLAIR) images for each patient. Infarct regions were segmented on the DWI images, and the cerebrospinal fluid regions were segmented on the FLAIR images (CSF_FLAIR_). The FLAIR images were overlaid onto the DWI images, and the cerebrospinal fluid regions on DWI images (CSF_DWI_) were segmented on the registered DWI images. One thousand three hundred and sixteen features were extracted from DWI volumes of interest (VOIs), CSF_FLAIR_ VOIs and CSF_DWI_ VOIs, respectively.

All DWI and FLAIR images of the AIS patients were evaluated by 2 board-certified neuroradiologists (radiologist 1, 8 years of experience in neuroradiology, radiologist 2, 10 years of experience in neuroradiology), and patients with DWI or FLAIR sequence with severe artifacts were excluded. Image registration between the DWI and FLAIR images was performed using the optimized automatic 3D registration tool in MIPAV.^1^ The infarct volumes (high-intensity signal on DWI images and apparent diffusion coefficient (ADC) < 620 × 10^3^ mm^2^/s) were drawn as DWI volumes of interest (VOI) using ITK-SNAP^2^ by the aforementioned neuroradiologists (radiologist 1) and checked by the radiologist 2 (Figure 2). The CSF region was segmented using the segmentation module in SPM12 software based on the FLAIR images. Statistical probability mapping was performed on the CSF images. Then, voxels with a probability > 0.6 were included as the final CSF region (CSF_FLAIR_ VOI). The VOI was then transformed into DWI space from FLAIR space using the transform matrix calculated by registering the FLAIR image to the DWI image (CSF_DWI_ VOI) (Figure 2). To confirm that the anatomical location of CSF was correct, the radiologist 2 checked the location visually using ITK-SNAP software by imposing the transformed CSF VOI on the DWI image.

### Image Feature Extraction and Selection

The radiomic features from DWI VOIs, CSF_FLAIR_ VOIs, and CSF_DWI_ VOIs were computed using PyRadiomics software (version: 3.0.1).^3^ PyRadiomics software complied with ISBI guidelines. The feature classes contained first-order features, shape features, and texture features. Finally, 1,316 features were extracted from each VOI. Before the analyses, variables with zero variance were excluded. Then, the missing values and outlier values were replaced by the median. Finally, the feature value was normalized to a standard normal distribution with μ = 0 and σ = 1.

Features of DWI VOIs and CSF_DWI_ VOIs, DWI VOIs, and CSF_FLAIR_ VOIs were screened separately. Considering the redundancy of the features and to reduce model overfitting, feature selection was performed using the enumeration method. In our study cohort, 70 positive cases were included; thus, the upper limit of the number of selected features should be less than 10% of the total number of positive cases. The minimum redundancy maximum relevance (MRMR) feature selection method was used to obtain 4 feature subsets with 5, 10, 15, and 20 features. Each subset was used to construct the predictive models using 7 machine learning methods (the 7 ML models were described in the “Machine Learning Model” section), the mean AUCs of the 7 models were measured, and the subset with the highest area under the curve (AUC) was chosen as the final feature subset.

### Machine Learning Model

The machine learning model was built from the established optimal feature subset of the training dataset. The hyperparameters were automatically selected by the search method. Seven machine learning methods for edema prediction (non-edema or edema): random forest (RF), support vector machine with kernel (svmLinear) or radial basis function kernel (svmRadial), Bayesian (Bayes), and k-nearest neighbor (KNN), adaptive boosting (Adaboost), and a neural network were constructed and compared. All training processes were performed in R software with the caret package, and the computation time of each ML model was approximately 5–10 min. Nested cross-validation was carried out to train the models with different ML methods. The outer cross-validation used the leave group out cross-validation (LGOCV) method, which repeatedly split the dataset 1 into 2 subsets, an analysis subset and an assessment subset at a ratio of 7:3. The analysis subset was used to train the model and the assessment subset was used to validate the model, and 100 times was adopted. The inner cross-validation used 10-fold cross-validation and was used in the analysis subset to tune the optimized hyperparameters and to construct the predictive model. Finally, each ML method was used to construct 100 models, and evaluate 100 AUC values and other metrics. The relative standard deviations (RSDs) were calculated using the following formula:


$$\mathrm{RSD}\%=\sigma_{\text{AUC}}/\mu_{\text{AUC}}\times 100\%,$$

where σ_AUC_ is the standard deviation of 100 AUC values and μ_AUC_ is the mean of the 100 AUC values. The smaller the RSD% is, the more stable the model is.

### External Validation

After the aforementioned process, a final model with comparable performance and stability was chosen and validated in the external validation set (Dataset 2).

### Statistical Analyses

Receiver operating characteristic (ROC) curve and precision-recall curve (PRC) were generated to evaluate the performances of the machine learning models, and the area under the ROC curve (AUC), the area under the PRC curve (auPRC), accuracy, sensitivity, specificity, negative predictive value (NPV), positive predictive value (PPV), precision and recall were calculated. All statistical analyses for the present study were performed with R 4.0.3. All statistical tests were two-sided, with a significance level of 0.05. The differences in performances of DWI + CSF_FLAIR_ model and DWI + CSF_DWI_ model revealed by ROC analysis and PRC analysis were evaluated according to Delong et al. (1988).

## Results

### Subject Classification

Of the 212 enrolled patients from dataset 1, 68 patients developed edema on follow-up CT. Dataset 2 consisted of 60 patients in the independent external validation set, and 22 patients developed edema follow-up CT. There were no significant differences in sex, age, NIHSS scores at admission, time from symptom onset to MRI, time from symptom onset to IVT, time from symptom onset to EVT and other clinical variables between edema group and non-edema group both in dataset 1 and dataset 2 (*P* > 0.05) (Table 1).

**TABLE 1:** Baseline characteristics of the study population.

Variables	Dataset 1 (Training set; *n* = 212)		*P-*value	Dataset 2 (Validation set; *n* = 60)		*P*-value
	Edema group	Non-edema group		Edema group	Non-edema group	
	(*n* = 68)	(*n* = 144)		(*n* = 22)	(*n* = 38)	
Sex (male), *n* (%)	44 (64.71%)	83 (57.64%)	0.327	12 (54.55%)	19 (50.00%)	0.734
Age, mean (SD), year	68.24 ± 15.38	67.41 ± 11.29	0.812	70.15 ± 12.34	68.65 ± 12.37	0.654
Time from symptom to MRI, mean (SD), min	213.81 ± 66.25	204.15 ± 79.59	0.411	229.43 ± 114.95	221.38 ± 109.20	0.793
Time from symptom to IVT, mean (SD), min	189.65 ± 99.47	178.32 ± 106.27	0.436	206.63 ± 103.44	197.55 ± 107.25	0.496
Time from symptom to EVT, mean (SD), min	256.73 ± 103.59	246.11 ± 112.48	0.314	267.34 ± 112.34	250.27 ± 102.08	0.355
NIHSS at admission, mean (SD)	13.80 ± 6.14	12.11 ± 7.02	0.380	14.211 ± 6.25	13.21 ± 6.88	0.315
Diabetes mellitus, *n* (%)	19 (27.94%)	25 (17.36%)	0.076	5 (22.73%)	6 (15.79%)	0.503
Hypertension, *n* (%)	59(86.76%)	109 (75.69%)	0.064	18 (81.82%)	29 (76.32%)	0.618
Atrial fibrillation, *n* (%)	26 (38.24%)	39 (27.08%)	0.100	6 (27.27%)	9 (23.68%)	0.757
Hyperlipidemia, *n* (%)	6 (8.82%)	8 (5.56%)	0.371	2 (9.09%)	2 (5.26%)	0.971
Therapy, *n* (%)			0.815			0.592
EVT	38 (55.88%)	78 (54.17%)		12 (54.55%)	18 (47.37%)	
IVT + EVT	30 (44.12%)	66 (45.83%)		10 (50.00%)	20 (52.63%)	

*NIHSS, National Institutes of Health Stroke Scale; EVT, endovascular thrombectomy; IVT, intravenous thrombolysis.*

### Feature Selection

**FIGURE 3:**
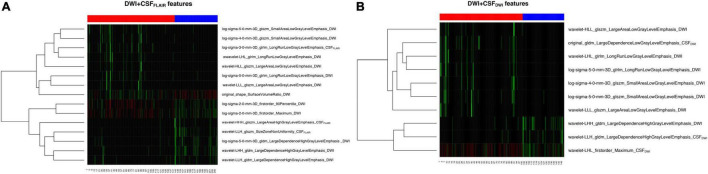
Heatmaps of the selected 15 features **(A)** and 10 features **(B)**.

The feature subset with 15 features based on DWI + CSF_FLAIR_ and the feature subset with 10 features based on DWI + CSF_DWI_ gained the highest mean AUC value and were assigned as the final feature subset to establish the different predictive models with different ML methods. The heatmaps in the cohort are shown in Figure 3; the heatmaps include 12 DWI features and 3 CSF_FLAIR_ features in DWI + CSF_FLAIR_ features (Figure 3A) and 7 DWI features and 3 CSF_DWI_ features in DWI + CSF_DWI_ features (Figure 3B).

### Performance and Stability of the 7 Machine Learning Models Based on DWI + CSF_FLAIR_ and DWI + CSF_DWI_

**FIGURE 4:**
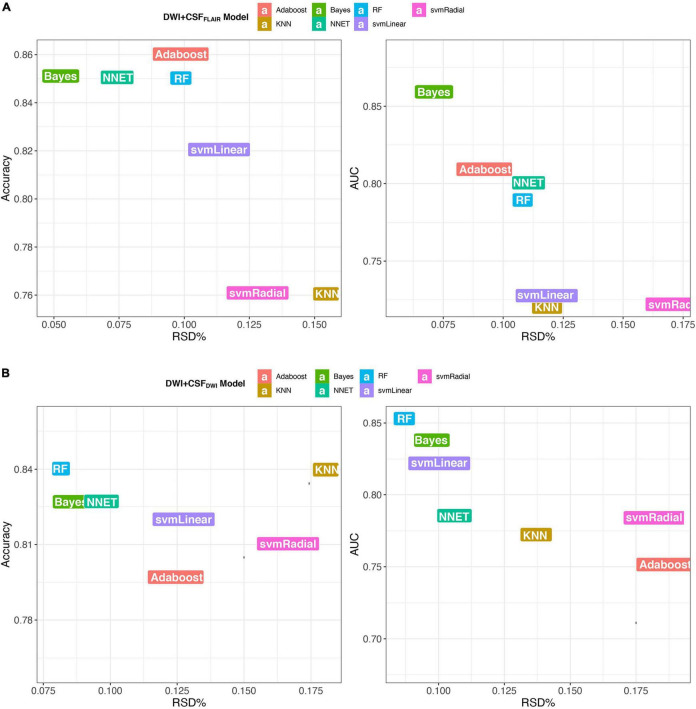
Accuracy and AUC vs. RSD of the 7 models, **(A)** models based on DWI + CSF_FLAIR_ features; **(B)** models based on DWI + CSF_DWI_ features.

In the training set, the 7 machine learning methods used the selected features to train the predictive models using nested cross-validation. The specified values are shown in Table 2. The Bayes model based on DWI + CSF_FLAIR_ had the highest AUC value and accuracy (AUC: 0.86; accuracy: 0.85; precision: 0.80; recall: 0.88), and the most stability (RSD% in AUC: 0.07, RSD% in accuracy: 0.05) of all the models (Figure 4A). The RF model based on DWI + CSF_DWI_ had the highest AUC value and accuracy (AUC: 0.86; accuracy: 0.84; precision: 0.79; recall: 0.84) and the most stability (RSD% in AUC: 0.09, RSD% in accuracy: 0.08) of all the models (Figure 4B). After comparing the performance and stability, the Bayes model based on DWI + CSF_FLAIR_ and the RF model based on DWI + CSF_DWI_ were chosen as the final models, respectively.

**TABLE 2:** The performance metrics of 7 models built with 7machine learning methods.

Model	ML methods	AUC	Accuracy	Sensitivity	Specificity	NPV	PPV	Precision	Recall
DWI + CSF_FLAIR_	Adaboost	0.81	0.86	0.82	0.83	0.91	0.81	0.82	0.88
	KNN	0.72	0.76	0.82	0.80	0.91	0.70	0.82	0.73
	Bayes	0.86	0.85	0.80	0.85	0.91	0.83	0.80	0.88
	NNET	0.80	0.85	0.84	0.84	0.92	0.80	0.84	0.86
	RF	0.78	0.85	0.86	0.85	0.93	0.78	0.86	0.85
	svmRadial	0.72	0.76	0.86	0.82	0.83	0.71	0.70	0.79
	svmLinear	0.73	0.82	0.86	0.82	0.92	0.73	0.86	0.80
DWI + CSF_DWI_	Adaboost	0.75	0.80	0.86	0.82	0.93	0.70	0.86	0.77
	KNN	0.76	0.84	0.76	0.79	0.88	0.75	0.76	0.80
	Bayes	0.84	0.83	0.87	0.81	0.87	0.75	0.76	0.80
	NNET	0.78	0.83	0.74	0.81	0.87	0.78	0.74	0.88
	RF	0.86	0.84	0.85	0.86	0.90	0.80	0.79	0.84
	svmRadial	0.78	0.81	0.71	0.77	0.87	0.77	0.71	0.87
	svmLinear	0.79	0.82	0.79	0.81	0.87	0.79	0.71	0.87

*KNN, k-nearest neighbor; NNET, neural network; RF, random forest; AUC, area under curve; NPV, negative predictive value; PPV, positive predictive value.*

### Independent External Validation

**FIGURE 5:**
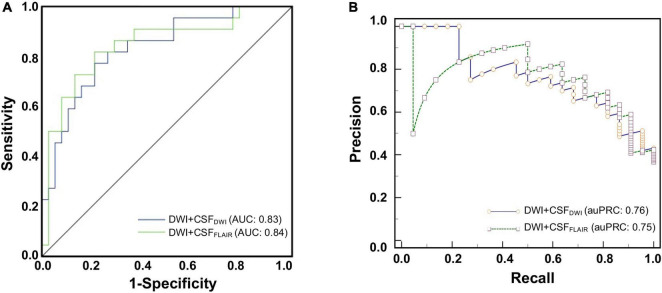
The ROC curves **(A)** and the precision-recall curves (PRCs) **(B)** of the Bayes model based on DWI + CSF_FLAIR_ features and the RF model based on DWI + CSF_DWI_ features from external validation for predicting edema after acute stroke.

The final models were validated in an independent external validation set. The ROC curves and PRCs of the validation set are shown in Figure 5. The AUC of the Bayes model based on DWI + CSF_FLAIR_ was 0.84, and the accuracy, sensitivity and specificity were 0.77, 087, and 0.78, respectively. The AUC of the RF model based on DWI + CSF_DWI_ was 0.83, and the accuracy, sensitivity and specificity were 0.81, 0.81, and 0.74, respectively. The auPRC of the Bayes model based on DWI + CSF_FLAIR_ was 0.75 with precision of 0.87 and recall of 0.75; the auPRC of the RF model based on DWI + CSF_DWI_ was 0.76 with precision of 0.81 and recall of 0.72. The performances of DWI + CSF_FLAIR_ model and DWI + CSF_DWI_ model showed no significantly differences (*P* > 0.05). The parameters are displayed in Table 3.

**TABLE 3:** The performance metrics of external validation.

Model	ML methods	AUC	Accuracy	Sensitivity	Specificity	NPV	PPV	Precision	Recall
DWI + CSF_FLAIR_	Bayes	0.84	0.77	0.87	0.78	0.87	0.78	0.87	0.75
DWI + CSF_DWI_	RF	0.83	0.81	0.81	0.74	0.81	0.75	0.81	0.72
*P*-value		0.879	0.483	0.811	0.872	0.823	0.848	0.771	0.763

*DWI, diffusion weighted imaging; FLAIR, fluid attenuated inversion recovery; AUC, area under curve; NPV, negative predictive value; PPV, positive predictive value.*

## Discussion

This study showed that an automated system developed using ML methods can be useful in predicting edema after acute stroke. We found that the feature subset with 15 features based on DWI + CSF_FLAIR_ and the feature subset with 10 features based on DWI + CSF_DWI_ were associated with edema. The Bayes model based on DWI + CSF_FLAIR_ and the RF model based on DWI + CSF_DWI_ had the highest performance and the most stability, respectively. Moreover, both models showed the same good generalization ability in external verification. Thus, our findings suggested that the ML approaches based on MRI radiomics features from infarction and CSF are valid and capable of being used to predict the edema after acute stroke patients in the clinic.

The cerebral edema after stroke is associated with poor prognosis and high mortality, and decreased consciousness, nausea or vomiting are the main clinical factors associated (Arboix et al., 2015). It is very important to early and accurate predict the risk of cerebral edema for improving the outcome. Our methods are different from previous image processing studies of edema, which have largely focused on CSF segmentation using CT. Chen et al. (2016) developed and validated an accurate automated approach to segment CSF on serial CT scans via integration of RF-based ML with geodesic active contour segmentation. Dhar et al. (2018) created an ML algorithm capable of segmenting and measuring CSF volume from serial CT scans of stroke patients to predict edema. This study aimed to predict edema by taking advantage of the radiomics features of CSF and infarct area on MRI scans. Our approach shares some similarity to previous approaches that automatically segment CSF (Chen et al., 2016; Dhar et al., 2018) using the ML approach. CSF segmentation in CT scans of stroke patients is a more complex technique than finding an optimal threshold (Chen et al., 2016). Intraclass correlation coefficients of 0.91 were observed for CSF segmentation accuracy in FLAIR images (Meier et al., 2018). Likewise, we also adopted automatic CSF segmentation in FLAIR scans in our study. Because of the low resolution of DWI scans, we did not directly segment the CSF, but registered the FLAIR images to DWI images and transformed the CSF_FLAIR_ space into DWI space using the transform matrix.

In this study, we focused on edema prediction rather than segmentation of CSF. ΔCSF has been suggested to be closely related to the prediction of malignant edema. Some research has shown that larger parenchymal hypoattenuation on CT is a reliable early predictor for malignant edema (Wu et al., 2018), and the signs of acute infarct on CT scans were independent predictors for all edema types (Kamel et al., 2020). Foroushani et al. (2020) found that incorporating quantitative CT-based imaging features (ΔCSF, infarct-related hypodensity volume, etc.) from baseline and 24 h CT enhanced the identification of patients with malignant edema. It is known that CT has limited ability to measure the size of early infarcts and MRI (DWI) has higher sensitivity for identifying acute ischemic lesions (Dibiasio et al., 2019). Therefore, we used a combination of CSF and infarct area on MRI scans to predict edema. However, it was difficult to qualify ΔCSF on MRI scans because of few patients had MRI scans both before and after therapy. Our study only predicted edema based on CSF and infarct area before therapy, which is also one of the limitations of this study.

In our study, we found that the feature subset with 15 features based on DWI + CSF_FLAIR_ and the feature subset with 10 features based on DWI + CSF_DWI_ were associated with edema, which including shape features, first order features and texture features. Shape features and first order features can indirectly reflect the infarct volume.

Texture features refer to the presentation of textures exhibited by special defined gray level matrices and each type of gray level matrix reflects certain aspects of the image. We found that both DWI and CSF texture features were closely related to edema, and DWI texture features contributed highly to our predictive model, including 9 DWI texture features of DWI + CSF_FLAIR_ model [4 gray level size zone matrix (GLSZM), 3 gray level dependence matrix (GLDM), 2 gray level run length matrix (GLRLM)] and 7 DWI texture features of DWI + CSF_DWI_ model (4 GLSZM, 2 GLRLM, 1 GLDM). The texture features indicated that infarction and CSF images in patients with non-edema had more homogeneity and lower contrast adjacent voxel relationships than those of edema, which cannot judgment by the naked eye. Further, we established and validated a stable ML model based on these features to predict edema after EVT in acute stroke patients. The 7 ML models in our study have been proven to have good prediction or classification performance in the medical imaging (Erickson et al., 2017; Kim et al., 2020; Shukla et al., 2020). We demonstrated that the Bayes model based on DWI + CSF_FLAIR_ and the RF model based on DWI + CSF_DWI_ had the highest AUC value and the most stability, respectively. RF analysis can automatically perform the task of feature selection and can provide a reliable feature importance estimate (Hashimoto et al., 2020; Hanko et al., 2021). Bayesian classification is a probabilistic approach to learning and inference based on a different view of what it means to learn from data; this form of classification allows for both modeling of uncertainty and updating or learning repeatedly as new data are made available (Hughes et al., 2017; Hackenberger, 2019). Du et al. (2020) found that the AUC-ROC value of the nomogram was 0.805 based on age, baseline NIHSS, blood glucose level, collateral circulation and recanalization in stroke patients treated EVT. Compared to previous studies on prediction of edema, ours has the following advantages: the same high prediction performances can be obtained only based on pretherapy DWI images, and clinicians could quickly evaluate the risk of edema before EVT therapy for stroke patients. After we chosen the final model, we validated the model in an independent external validation set. The AUCs of the model were similar to those of the training set. In addition, we compared the performance of the ML model with different modalities (DWI + CSF_DWI_ vs. DWI + CSF_FLAIR_), and the results showed that the AUCs and auPRCs did not differ significantly between the two models. The results showed that although the resolution of DWI was low, the prediction performance of CSF_DWI_ segmentation was the same as that of CSF_FLAIR_ segmentation.

The present study has several limitations. Firstly, the sample size was relatively small, and the non-edema and edema data was unbalanced. However, it should be noted that the outlier of the median was used to obtain balanced data and reliable results. Secondly, ΔCSF and clinical data were not included for edema prediction. Thirdly, a large number of patients with accurate CSF_FLAIR_ segmentation and inaccurate CSF_DWI_ segmentation were excluded. The segmentation method of CSF_DWI_ still needs further research to improve the accuracy. In addition, the VOI segmentation in our study were done manually which limits the broad scalable implementation of this approach. In fact, we have been studying the automatic segmentation approach for DWI lesions of AIS, and also have made some progress (Zhu et al., 2021). In the next study, we will attempt to predict the edema using an automatically segmentation approach to evaluate its wide clinical applicability. Finally, we recruited subjects from a large stroke cohort but had to limit our analysis to those patients who had EVT therapy. This likely biased our sample to patients with more severe strokes symptoms with a higher incidence of edema than a truly unselected cohort.

## Conclusion

In conclusion, we established and validated an automated ML system based on MRI radiomics features from infarction and CSF to predict edema after EVT for AIS. The MRI radiomics features from infarction and CSF may offer an affective imaging biomarker for predicting edema.

## Data Availability Statement

The raw data supporting the conclusions of this article will be made available by the authors, without undue reservation.

## Ethics Statement

The studies involving human participants were reviewed and approved by the Local Ethics Committee of Nanjing Medical University. The patients/participants provided their written informed consent to participate in this study. Written informed consent was obtained from the individual(s) for the publication of any potentially identifiable images or data included in this article.

## Author Contributions

LJ and CZ designed the experiment, collected the data, performed the analysis, and wrote the manuscript. SW, ZA, TS, HZ, and SD helped collect the data and perform the analysis. XY and Y-CC contributed to the discussion and manuscript revision. All authors read and approved the manuscript.

## Conflict of Interest

SD was employed by the company GE Healthcare. The remaining authors declare that the research was conducted in the absence of any commercial or financial relationships that could be construed as a potential conflict of interest.

## Publisher’s Note

All claims expressed in this article are solely those of the authors and do not necessarily represent those of their affiliated organizations, or those of the publisher, the editors and the reviewers. Any product that may be evaluated in this article, or claim that may be made by its manufacturer, is not guaranteed or endorsed by the publisher.

## Footnotes

1
http://mipav.cit.nih.gov

2
www.itksnap.org

3
https://pyradiomics.readthedocs.io/en/latest/
